# Evaluation of the Diagnostic Sensitivity of Digital Vibration Sensors Based on Capacitive MEMS Accelerometers

**DOI:** 10.3390/s24144463

**Published:** 2024-07-10

**Authors:** Marek Fidali, Damian Augustyn, Jakub Ochmann, Wojciech Uchman

**Affiliations:** 1 Department of Fundamentals of Machinery Design, Silesian University of Technology, Konarskiego 18A, 44-100 Gliwice, Poland; marek.fidali@polsl.pl (M.F.); damian.augustyn@polsl.pl (D.A.); 2 Department of Power Engineering and Turbomachinery, Silesian University of Technology, Konarskiego 18, 44-100 Gliwice, Poland; jakub.ochmann@polsl.pl

**Keywords:** MEMS accelerometer, vibration measurement, bearing faults, diagnostics, condition monitoring

## Abstract

In recent years, there has been an increasing use of digital vibration sensors that are based on capacitive MEMS accelerometers for machine vibration monitoring and diagnostics. These sensors simplify the design of monitoring and diagnostic systems, thus reducing implementation costs. However, it is important to understand how effective these digital sensors are in detecting rolling bearing faults. This article describes a method for determining the diagnostic sensitivity of diagnostic parameters provided by commercially available vibration sensors based on MEMS accelerometers. Experimental tests were conducted in laboratory conditions, during which vibrations from 11 healthy and faulty rolling bearings were measured using two commercial vibration sensors based on MEMS accelerometers and a piezoelectric accelerometer as a reference sensor. The results showed that the diagnostic sensitivity of the parameters depends on the upper-frequency band limit of the sensors, and the parameters most sensitive to the typical fatigue faults of rolling bearings are the peak and peak-to-peak amplitudes of vibration acceleration. Despite having a lower upper-frequency range compared to the piezoelectric accelerometer, the commercial vibration sensors were found to be sensitive to rolling bearing faults and can be successfully used in continuous monitoring and diagnostics systems for machines.

## 1. Introduction

Rolling bearings are used in almost every type of rotating machinery. Most machine breakdowns relate to bearing failures; thus, it is very important to diagnose bearing conditions and predict the moment of failure occurrence [1,2,3]. Many bearings fail prematurely due to contamination, poor lubrication, misalignment, temperature extremes, poor fitting/fits, shaft unbalance, and misalignment [4]. The occurrence of bearing faults leads to an increase in the bearing vibration; therefore, in diagnosing the condition of rolling bearings, measurements and analysis of vibration signals are most often used [3].

### 1.1. Vibration Symptoms of Bearing Faults

**Figure 1:**
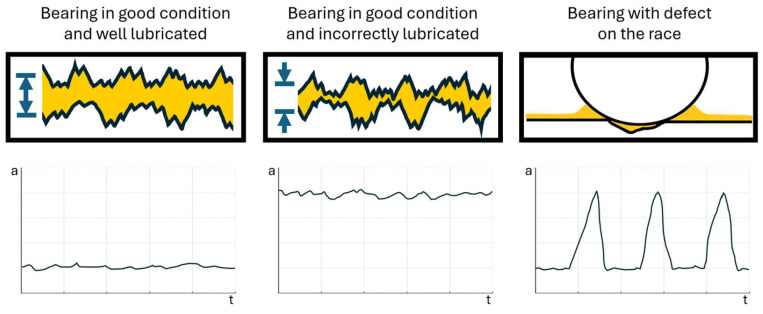
The influence of the surface conditions of interacting bearing elements on the number and intensity of generated vibration signals.

Effective diagnosis of rolling bearings based on vibration measurements first requires an understanding of the relationship between how damage occurs in bearings and the symptoms of that damage visible in vibration signals [5,6]. A bearing consists of rolling elements mounted in a cage and rolling on an inner and outer race. If we take a closer look at the contact area (Figure 1), in an efficient and well-lubricated bearing, the rolling elements are separated from the race surface by a layer of grease such that during the rolling, only the highest peaks of surface roughness will interfere with each other, generating hundreds of small-amplitude short pulses [7]. Due to the random distribution of the roughness, the pulses generated will have the character of random noise. It can therefore be concluded that a healthy bearing is a random noise generator. As the lubrication conditions deteriorate and the lubrication film thickness is gradually reduced, the pulses generated will be more intense, so the level of perceived noise will be higher.

**Figure 2:**
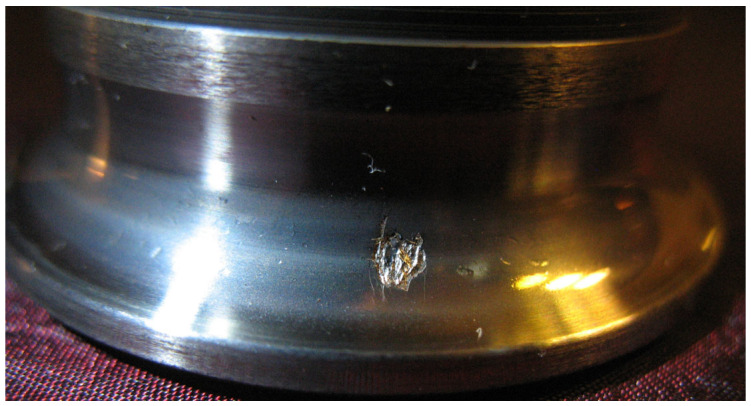
Material loss on the inner race of the bearing.

In an operating bearing, as a result of various wear mechanisms, including, but not limited to, fatigue wear, a small loss of material may develop on the surface of one of the raceways (Figure 2), causing each of the rolling elements to come into collision with the damage systematically, while generating cyclic pulses with an amplitude exceeding the noise level by up to 1000 times.

The frequency and intensity of the pulses will strongly depend on the bearing geometry (number and diameter of rolling elements and bearing race diameters) and shaft speed. The characteristic frequencies of the pulses arising from the various bearing events can be calculated from the analytical relationships shown below. The rolling element pass frequency over the single defect on the outer-race BPFO (ball pass frequency, outer race) is defined as follows:

(1) $$\mathrm{BPFO}=\frac{{\mathrm{nf}}_{r}}{2}\left\{1-\frac{d}{D}\cos\phi \right\},$$

where n is the number of rolling elements, $f_{r}$ is the rotational frequency, d is the rolling element diameter, D is the pitch diameter, and ϕ is the angle of load. The ball pass frequency over the single defect on the inner race BPFI is defined as follows:

(2) $$\mathrm{BPFI}=\frac{{\mathrm{nf}}_{r}}{2}\left\{1+\frac{d}{D}\cos\phi \right\}.$$

The frequency related to the cage speed FTF (fundamental train frequency) is defined as follows:

(3) $$\mathrm{FTF}=\frac{f_{r}}{2}\left\{1-\frac{d}{D}\cos\phi \right\}.$$

The rolling element spin frequency $\mathrm{BSF}\left(\mathrm{RSF}\right)$ is defined as follows:

(4) $$\mathrm{BSF}\left(\mathrm{RSF}\right)=\frac{D}{2d}\left\{1-{\left(\frac{d}{D}\cos\phi \right)}^{2}\right\}.$$

The impulses generated when rolling elements collide with a defect on one of the races are called shock or impact impulses and, due to the high stiffness of the elements involved in their generation, are characterized by a very short duration of a few to tens of microseconds. The pulses generate elastic waves in the material, which propagate at a speed of around 5000 m/s in steel. Furthermore, due to the short pulse duration, in the spectrum, the pulse energy is distributed over a very broad frequency band beyond 40 kHz [8].

The bearing is not an isolated component but cooperates with the shaft and, additionally, carries loads from, among other things, forces generated by the residual imbalance of the rotor and/or shaft misalignment. Consequently, the shock pulses generated during the initial stage of bearing degradation are very weak in relation to the signal components generated by inertia forces. It follows that in order to detect bearing damage at an early stage, it is useful to measure vibrations over a wide frequency band, covering the ultrasonic range, and in order to extract weak pulses caused by mechanical damage from the broadband signal, it is necessary to use appropriate methods for vibration signal processing and analysis.

### 1.2. Methods of Bearing Diagnostics

The condition of rolling bearings can be assessed by the results of diagnostic tests using temperature measurements, lubricant tests, thrust torque measurements, ultrasonic measurements, and vibration and noise measurements [2].

Due to the high availability of test equipment, vibration measurement and analysis is one of the more frequently used methods for diagnosing rolling element bearings. In the field of vibration signal analysis for rolling element bearing condition assessment, more or less sophisticated methods of signal analysis and evaluation based on the Hilbert transform and analysis of the vibration acceleration envelope signals in the time and frequency domains are used [1]. Over the years, a number of commercial solutions have been developed for rolling bearing diagnostics, such as the SPM (shock pulse method) and SPM HD (shock pulse method (higher definition)) from the SPM Instrument AB, the spike energy spectrum (gSE) from Rockwell Automation/ENTEK and PeakVue from CSI/Emerson, SEE (spectral emitted energy) and AEE (acoustic emission enveloping), ENV Acc and HFD from SK, and the BCU (bearing condition unit) from Schenck, among others. As industrial practice shows, the condition assessment of rolling element bearings is often based on basic numeric estimators of vibration acceleration signals after prior high-pass filtering and subsequent observation of time series as a function of operating time and trend analysis. The most commonly determined vibration acceleration signal amplitude estimators (signal features) are the peak value (aPeak) and/or the rms value (aRMS). Using numerical parameters, it is also possible to refer to limit values defined in the standards or practical diagnostic recommendations developed by diagnosticians or some companies [9]. An example of a standard which defines the criteria for evaluating the bearing condition based on point features determined from a broadband vibration acceleration signal is ISO 13373-3 [10].

The values of rms and peak amplitudes can also be used to determine the dimensionless parameter, like crest factor (Equation (5)). This represents the ratio of the peak value of the vibration signal to its RMS value in a given vibration frequency range. If the crest factor increases, the rolling bearing deteriorates. However, in the last phase of damage, the value of the peak factor may decrease. Therefore, this ratio should be used from the beginning of the bearing’s life.

(5) $$C_{f}=\frac{\left|x_{Peak}\right|}{x_{RMS}}$$

where $x_{Peak}$ is the peak amplitude and $x_{RMS}$ is the effective amplitude.

Another parameter used for bearing defect detection is kurtosis. This statistical parameter (Equation (6)) describes the flatness of a Gaussian distribution, and, for a strictly random signal, its value equals 3.0. Because bearings in good condition theoretically, it should generate random noise, and kurtosis serves as an indicator of a healthy bearing. If mechanical degradation in the bearing begins, the kurtosis increases, and for deteriorated bearings, it can be higher than 10 or 15.

(6) $$K=\frac{\frac{1}{n}\Sigma_{i=1}^{n}{\left(x_{i}-\mu \right)}^{4}}{\sigma^{4}}-3$$

where $x_{i}$ is the *i*-th value of the feature, μ is the population mean, σ is the population standard deviation, and n is the sample size.

The usefulness of using numerical features in assessing the bearing condition is reflected in the VDI 3832 standard [11], which defines, among other things, a diagnostic parameter helpful for assessing the condition of rolling element bearings $K\left(t\right)$, also called the Sturm diagnostic coefficient. It is calculated according to Equation (7) from the product of the peak and rms values of the vibration accelerations in the frequency range from 1 to 10 kHz, which is related to the product of the reference rms and peak values of the vibration accelerations measured at the beginning of the bearing’s operation.

(7) $$K\left(t\right)=\frac{\mathrm{aRMS}\left(0\right)\cdot \mathrm{aPeak}\left(0\right)}{\mathrm{aRMS}\left(t\right)\cdot \mathrm{aPeak}\left(t\right)},$$

where $\mathrm{aRMS}\left(0\right)$ is the RMS for the start point in time, $\mathrm{aPeak}\left(0\right)$ is the maximum value for start, $\mathrm{aRMS}\left(t\right)$ is the current RMS, and $\mathrm{aPeak}\left(t\right)$ is the current maximum value.

The value of the parameter K(t) decreases with the deterioration of the bearing condition, making it possible to define the ranges of the limit values for the parameter and to relate them to the expected bearing condition. The limit values of the K(t) parameter are shown in Table 1.

Rolling bearing vibration diagnostic methods based on numerical parameters allow for the damage to be detected early enough that bearing replacement can be planned and carried out at the most convenient time for the production process and before potential failure.

### 1.3. Measuremnt of Bearing Vibration

Piezoelectric accelerometers (IEPE, ICP) have been used for many years in rolling bearing diagnostics, which, when connected to portable vibration meters or continuous monitoring systems, allow for the effective detection and identification of bearing damage at a very early stage [12]. Capacitive accelerometers, so-called MEMS accelerometers, have been on the market for a long time, alongside piezoelectric accelerometers [6,13,14].

Landi et al. [15] presented a prototype MEMS sensor accelerometer for monitoring vibrations over a wide frequency range. The research presented included a sensor calibration procedure and was carried out on an in-house test stand. Staszewski et al. [16] presented a MEMS vibration sensor design with a wide frequency range up to 10 kHz, which can replace traditional sensors due to high sensitivity, low noise, and lower costs. The sensor prototype tested on a rig with a faulty rolling element bearing demonstrated effectiveness in fault detection and comparable performance to a piezoelectric accelerometer. Zusman [17] presented a comparison of traditional piezoelectric and modern MEMS-based vibration sensors used in machinery condition monitoring and fault diagnostics. Experimental data and detailed comparisons of output noise level and spectrum density for several popular piezoelectric and MEMS vibration sensors are presented.

Rossi et al. [18] focused on demonstrating the sufficient accuracy of MEMS-based data monitoring compared to a reference, a conventional mini-integrated circuit piezoelectric (ICP). Investigating the vibration of turbofan engine fan blades, the MEMS was shown to have a satisfactory level of measurement accuracy of ±5% deviation with respect to the ICP at the angular velocity tested from 0 to 300 rpm. Varanis et al. [19] presented the use of MEMS sensors for measuring mechanical vibrations and a broad literature review on their use in various applications. Two experiments were also performed comparing the amplitudes and frequencies of oscillations measured by MEMS sensors and piezoelectric accelerometers in the time and frequency domains. Augustyn et al. [20] presented the results of research on the identification of the frequency characteristics of a digital MEMS accelerometer dedicated to monitoring the condition of machines. The specified characteristics indicate the possibility of using the sensor for basic machine diagnostics in accordance with the ISO 10816 [21] and ISO 20816 standards [22]; however, non-linearities at the limits of the measurement band may limit its use in precise scientific measurements. Anslow [23] presented the design of a mechanical housing for a MEMS accelerometer, which ensures high-quality vibration data for machine condition monitoring (CbM). This paper presents modal analysis, vibration sensor design guidelines, and housing design examples for single-axis and three-axis MEMS accelerometers, highlighting the importance of avoiding resonance and ensuring the appropriate housing natural frequency. However, Albarbar et al. [24] have shown experimentally that the selection of a suitable MEMS sensor is crucial for adequate monitoring of the desired quantity. In their study, they compared data obtained with sensors dedicated to measuring different types of signals: sinusoidal, random, and impulsive. They showed apparent differences between the results and suggested using one of the sensors for purposes other than monitoring the condition of the machine.

### 1.4. Contemporary Digital Vibration Sensors and Its Usefulness to Bearing Faults Diagnostics

With the advent of Industry 4.0 and IIoT technology, many automation companies are using MEMS accelerometers to build vibration sensors; they allow for vibration measurement and direct evaluation of vibration signals thanks to an integrated ADC and microcontroller [25,26,27].

These types of sensors have a digital output; thus, they can be called digital vibration sensors.

A digital vibration sensor can be considered as such if it has at least one of the following features:

- ADC converter and microcontroller.
- The ability to process and analyse measured signals.
- Ability to linearization of processing characteristics.
- Digital two-way communication interface.
- Self-test and auto calibration unit.
- The ability to learn and make independent decisions.

**Figure 3:**
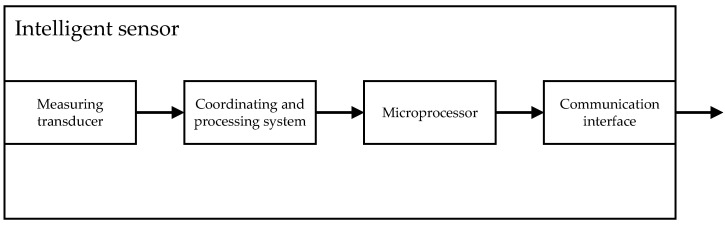
A block diagram of the digital accelerometer.

In order to be able to implement these functions, integrated in the sensor are a measuring transducer, a conditioning system, a microprocessor, and a communication interface which provides an estimation of vibration parameters and transfers it to the sensor registers; it can be read using popular data exchange protocols in automation, such as Modus RTU or IO Link. The use of this type of solution simplifies the design and implementation of continuous monitoring and diagnostic systems and facilitates data transfer to predictive and cloud systems. (Figure 3).

Commercial vibration sensors based on MEMS accelerometers have been available on the market for some time now, allowing for the measurement of a whole range of numerical parameters useful in machine diagnostics. Table 2 provides a comparison of the parameters of exemplary digital vibration sensors, while Table 3 summarizes the diagnostic parameters determined by the sensors and provided by the digital interface [25,26,27].

It is noticeable that the sensors make available the classic vibration parameters for assessing the overall condition of the machines based on the, e.g., ISO 20816 [28] standard. These include rms vibration velocity amplitudes (vRMS) measured in the 10–1000 Hz band. There are also standard parameters used in evaluating the condition of bearings, such as rms amplitudes (aRMS), peak (aPeak), and peak-to-peak amplitude (aPP) of vibration accelerations, which, depending on the sensor, are determined in the full available frequency band or can be determined in the high-frequency band above 1000 kHz. When analyzing the parameters of the above-presented vibration sensors, the frequency band does not exceed 4 kHz. Considering how bearing faults occur and how vibration signals are emitted, this range may not be sufficient in some cases.

Despite the low price of the aforementioned sensors and the simplicity of their implementation, the question arises as to how the vibration parameters determined by MEMS accelerometers are sensitive to bearing damage at different levels of severity. This paper attempts to answer this question by presenting the results of diagnostic sensitivity estimation for rolling element bearing measurements performed with the use of the two commercially available sensors with embedded MEMS accelerometers.

### 1.5. Novelty of This Research

The novelty of this study lies in its precise evaluation of the diagnostic sensitivity of digital vibration sensors based on MEMS accelerometers in detecting rolling bearing faults. Compared to previous studies, this approach stands out by directly comparing these sensors with traditional piezoelectric accelerometers in controlled laboratory conditions. A key finding is the effectiveness of MEMS sensors in detecting typical fatigue faults in bearings despite their lower-frequency bandwidth, making them a cost-effective alternative to piezoelectric vibration sensors.

## 2. Materials and Methods

### 2.1. The Test Bench and Experiment Description

**Figure 4:**
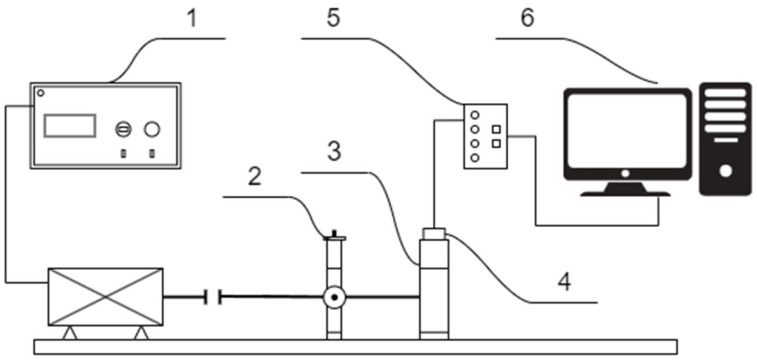
The test bench: 1—engine rotation speed controller; 2—radial load; 3—bearing housing; 4—vibration sensor with magnet holder; 5—processing electronics; 6—PC.

In order to assess the sensitivity of the diagnostic parameters determined by modern MEMS-based digital vibration sensors to bearing faults of different intensities and under different operating conditions, a series of active diagnostic experiments were planned and carried out on a test rig located at the Department of Fundamentals of Machinery Design of the Silesian University of Technology in Gliwice. The test stand consisted of a drive motor and a motor controller, allowing for rotational speed change; a bearing housing for mounting the tested bearings; and a loading system, allowing for radial load application to the tested bearing. The test rig was equipped with measurement systems to measure bearing housing vibrations using two commercial digital vibration sensors (SE1 and SE2) (see Table 2, items 1 and 2) and a piezoelectric accelerometer connected to an industrial programmable signal processing module. A PCB T352C34 (PCB Piezotronics, Inc., Depew, NY, USA) miniature piezoelectric accelerometer with a sensitivity of 100 mV/g and frequency range 0.5–10,000 Hz was used. A piezoelectric accelerometer was applied to collect reference data, which were used for comparison with data from the digital sensors being evaluated. The first tested sensor (SE1) was connected to the manufacturer’s dedicated measurement and data acquisition module, interfacing with the PC via a web browser. The second tested digital vibration sensor (SE2) was connected to a PC using a dedicated RS485-to-USB serial transmission converter. A script written in the MATLAB R2020b environment was used to acquire data from SE2. The piezoelectric vibration sensor was interfaced with processing electronics, also connected to a PC, which was equipped with dedicated software. All the sensors were mounted using a magnet holder. Figure 4 presents a diagram of the laboratory stand.

**Figure 5:**
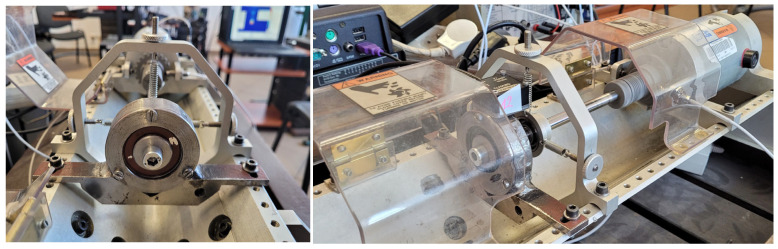
Experimental setup.

Figure 5 presents the experimental setup used for the research.

The tests were carried out on a set of 11 deep-groove ball bearings with polymer cage type 6303, 6 of which were brand new bearings that were considered to be in perfect condition. The new bearings were assigned identifiers N1–N6. The technical conditions of the remaining 5 bearings are characterized in Table 4.

For bearings D1–D4, the damage was introduced manually. In the case of bearing D4, progressive cage damage was simulated between measurements by cutting through the cage at selected points to finally remove it completely. For each bearing, vibration measurements were taken at three shaft speeds, 600 rpm, 1500 rpm, and 3000 rpm, and each was loaded with the same radial force.

For the piezoelectric sensor, the raw acceleration signal was recorded at a sampling rate of 100 kSamples/s for a period of 10 s in the full frequency range of 2–10,000 Hz. The collected signals were subjected to processing and analysis. Processing consisted of band-pass filtering in bands 10–10,000 Hz and 1000–10,000 Hz. The processed acceleration signals were segmented into time sub-realizations of 1 s duration and then analyzed to determine diagnostic parameters corresponding to those determined by the digital vibration sensors tested. Processing and analysis of the acceleration signals from the piezoelectric sensor were carried out in the Python computational environment.

### 2.2. Method of Evaluation of the Diagnostic Sensitivity of Investigated Digital Vibration Sensors

For the purposes of the described research, diagnostic sensitivity can be defined as a quantitative measurement of the relative change in the value of a diagnostic signal feature due to a small change in the technical condition of the diagnosed object [29,30,31]. It can be assumed that if a small change in the state causes a significant relative change in the value of the diagnostic parameter, we can speak of the parameter’s sensitivity to change. It can be assumed that the technical condition against which the changes of condition will be determined will be some reference condition; e.g., in the case of bearings, this is the good condition, characterized by the bearing at the beginning of operation. Therefore, assuming that the value of a specific characteristic of the diagnostic signal will be a measure of the current condition, the sensitivity measure $S_{p}$ can be defined according to the following equation:

(8) $$S_{p}\left(c_{i},t_{j}\right)=\left|1-\frac{p\left(c_{i},t_{j}\right)}{p\left(c_{0},t_{0}\right)}\right|\cdot 100\%,$$

where $c_{i}$ is the value of the characteristic technical condition at the moment of time $t_{j}$, $c_{0}$ is the reference value of the characteristic technical condition, and $t_{0}$ is the beginning of the object’s operation. In the considerations, the moment of time $t_{j}$ should be considered in the sense of operating time counted in hours, days, or months. In this case, the feature value can be estimated from the vibration signal over a short integration time interval, counted in milliseconds or seconds. It can be considered that if the sensitivity value meets the following condition, i.e.,$\;S_{p}\geq 25\%$, the change in the value of the characteristic is significant.

Due to the fact that two digital sensors and one piezoelectric sensor were used for the tests, we decided to compare the diagnostic sensitivities of the sensors in such a way that the differences between the sensitivities between the piezoelectric sensor and each of the digital sensors could be determined. For this purpose, a measurement of differential sensitivity (${\mathrm{DS}}_{\mathrm{SE}}$), defined as follows, was introduced:

(9) $${\mathrm{DS}}_{\mathrm{SE}}=S_{p_{n}}^{{\mathrm{SE}}_{\mathrm{ref}}}-S_{p_{n}}^{{\mathrm{SE}}_{i}}\;,$$

where $S_{p_{n}}$ is the diagnostic sensitivity of a given signal characteristic, ${\mathrm{SE}}_{\mathrm{ref}}$ is the reference sensor, and ${\mathrm{SE}}_{i}$ is the sensor under test. It can be assumed that if the value of ${\mathrm{DS}}_{\mathrm{SE}}$ meets the following condition, i.e., ${\mathrm{DS}}_{\mathrm{SE}}\geq 25\%$, then the difference in the way the value of the characteristic is estimated by the sensor under test is significant.

The data collected during the experiments was processed, ordered, and analyzed. As the feature values for bearings in good condition will serve as reference values, the quality of the collected feature values of the vibration signals for bearings in good condition was assessed first. The coefficient of variation (CV), determined as follows, was used to assess the quality of the data:

(10) $$\mathrm{CV}=\frac{\sigma }{\mu }\cdot 100\%,$$

where σ is standard deviation and μ is a mean value of signal feature value.

## 3. Results

### The Test Bench and Experiment Description

The CV values determined by Equation (8) are shown in Table 5.

The coefficient of variation for the new bearings in the case of the piezoelectric sensor took values lower than or close to the coefficients of variation of the digital sensors. A deviation can be seen for the kurtosis and CF parameters, where the CV index values were higher for the piezoelectric sensor at rotational speeds of 1500 and 3000. This behavior should be explained by the higher standard deviations resulting from the wider frequency range for which the signal features were determined.

**Figure 6:**
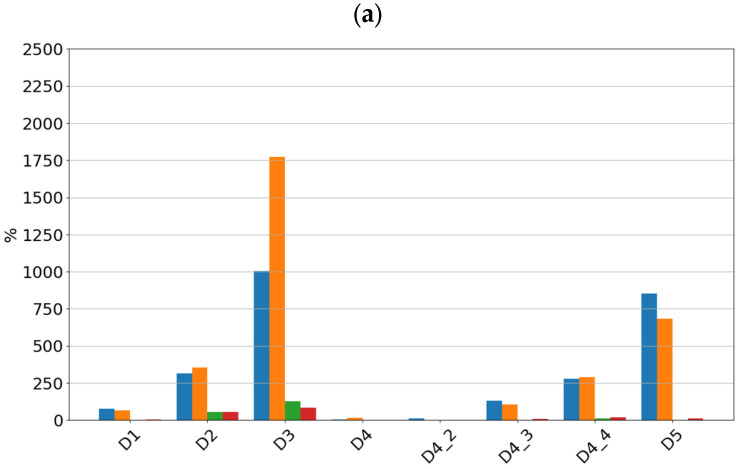

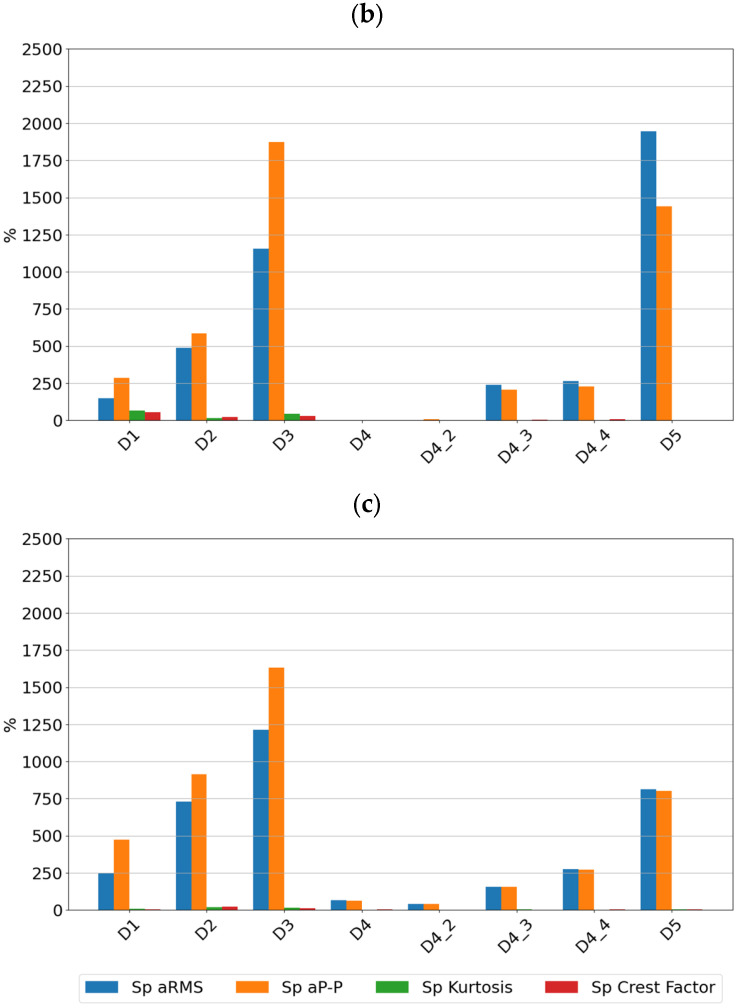
Sensitivity value of features for sensor SE1 at speeds of (**a**) 600 rpm, (**b**) 1500 rpm, and (**c**) 3000 rpm.

**Figure 7:**
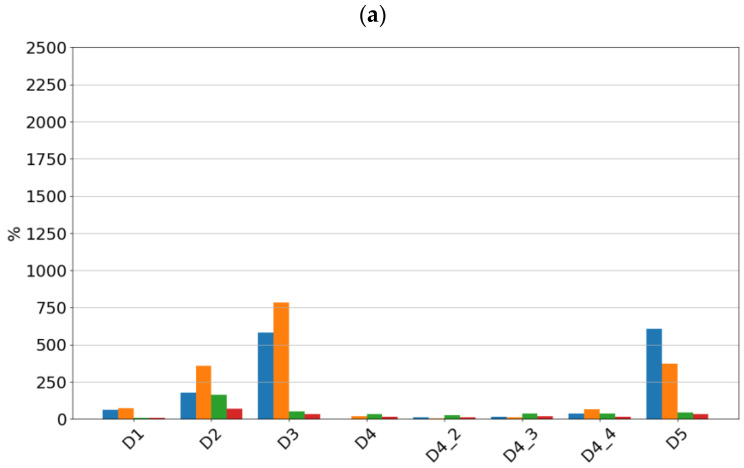

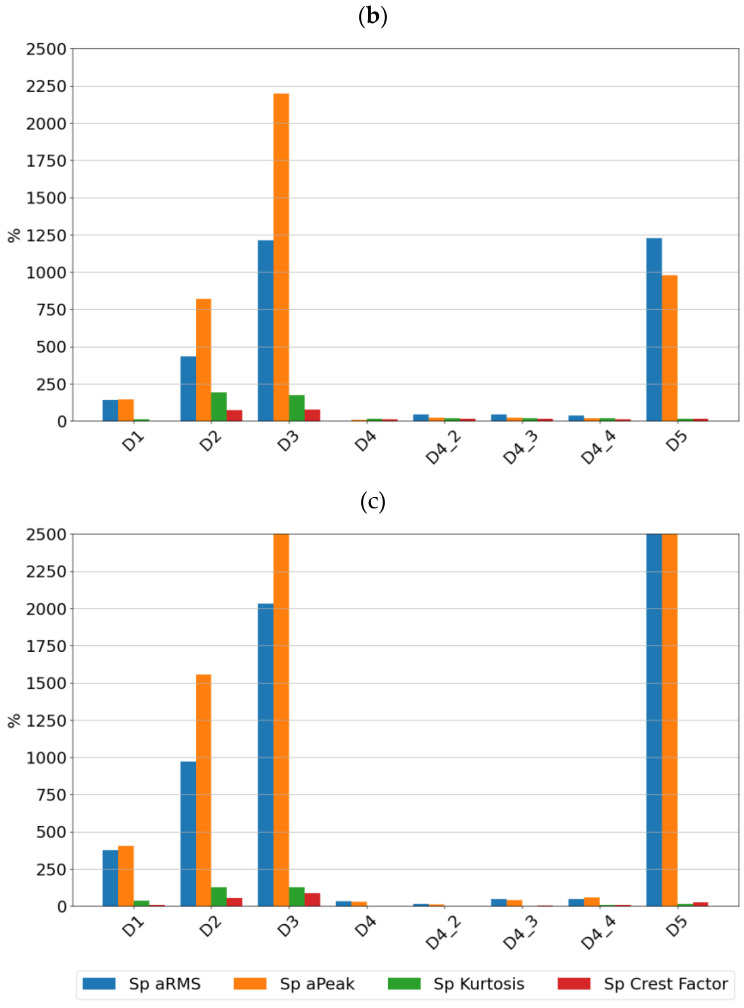
Sensitivity value of features for sensor SE2 at speeds of (**a**) 600 rpm, (**b**) 1500 rpm, and (**c**) 3000 rpm.

For each of the determined features of the vibration signals, sensitivity values were determined, which are summarized in Figure 6 for sensor SE1 and Figure 7 for sensor SE2.

**Figure 8:**
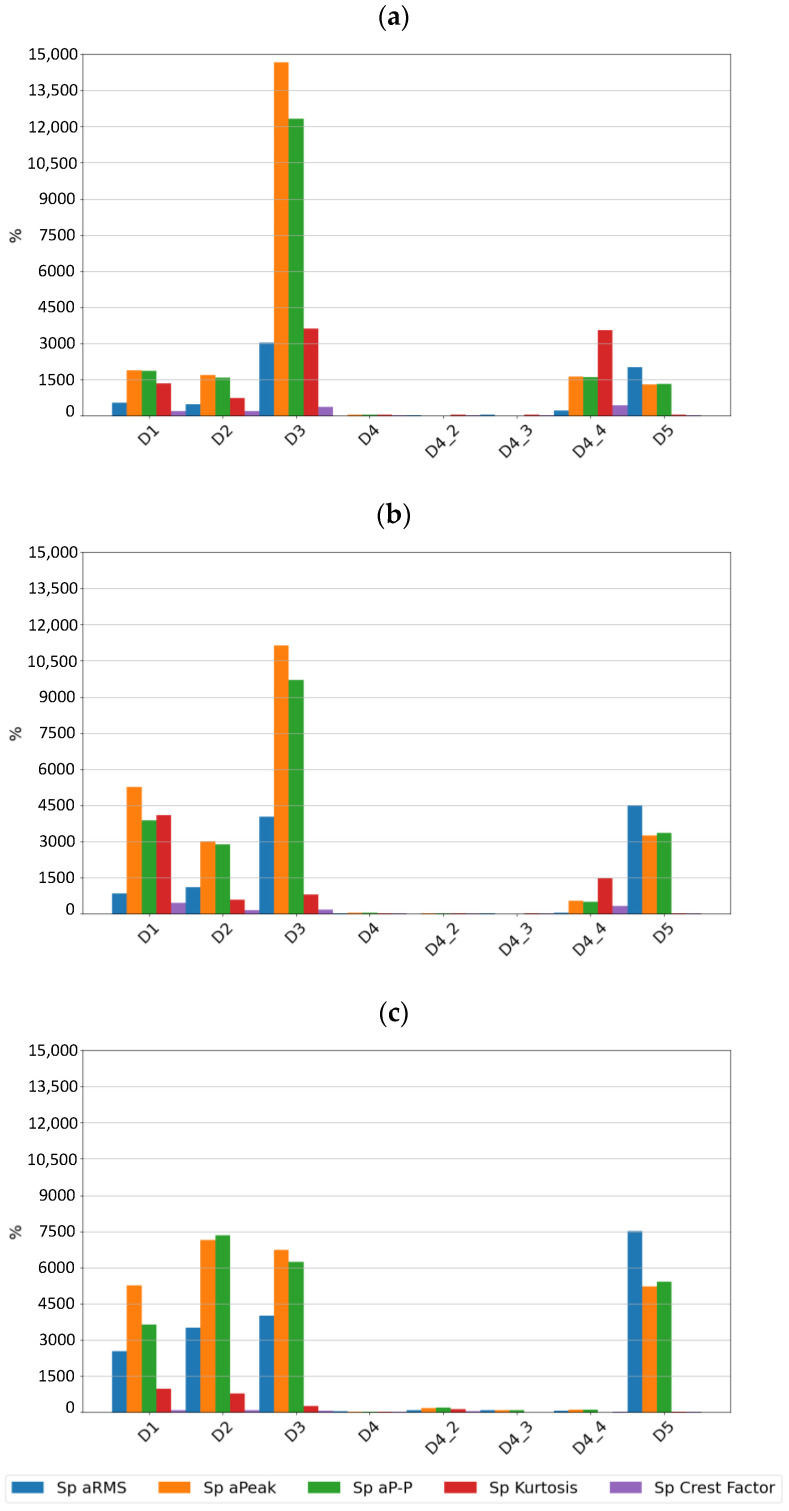
Sensitivity value of features for sensor SEref at speeds of (**a**) 600 rpm, (**b**) 1500 rpm, and (**c**) 3000 rpm.

For the piezoelectric accelerometer, the sensitivity values are presented in Figure 8.

By analyzing the sensitivity values determined, it can be seen that the tested sensors SE1 and SE2 show very high sensitivity to typical bearing damages. Both sensors showed the highest sensitivity to the rolling element defect, followed by damage to the inner race, damage to the outer race, and lack of lubrication. From a diagnostic parameters point of view, peak and peak-to-peak values showed the highest sensitivity, although, in the absence of lubrication, the rms amplitude showed the highest sensitivity. It is worth noting that in the non-lubricated case, the sensitivity increased with rotational speed and the highest values were achieved for the features determined from the high-pass filtered signal. For all features for the sensors considered, the lowest sensitivity was observed for cage damage. This is a specific kind of damage manifesting itself with different symptoms depending on its intensity, the design of the cage, and the material from which it is made. From a spectrum analysis perspective, the cage damage manifests itself with a frequency component equal to 0.4f_n_, where f_n_ is the rotational speed frequency. This makes it possible to see that the high-pass filtering of the signal in this case can make the detection of this damage more difficult. This phenomenon is apparent if we compare the feature values of the SE1 sensor, which were determined for the full sensor frequency range, and the feature values of the SE2 sensor, where were determined for the 1000–4000 Hz range.

**Figure 9:**
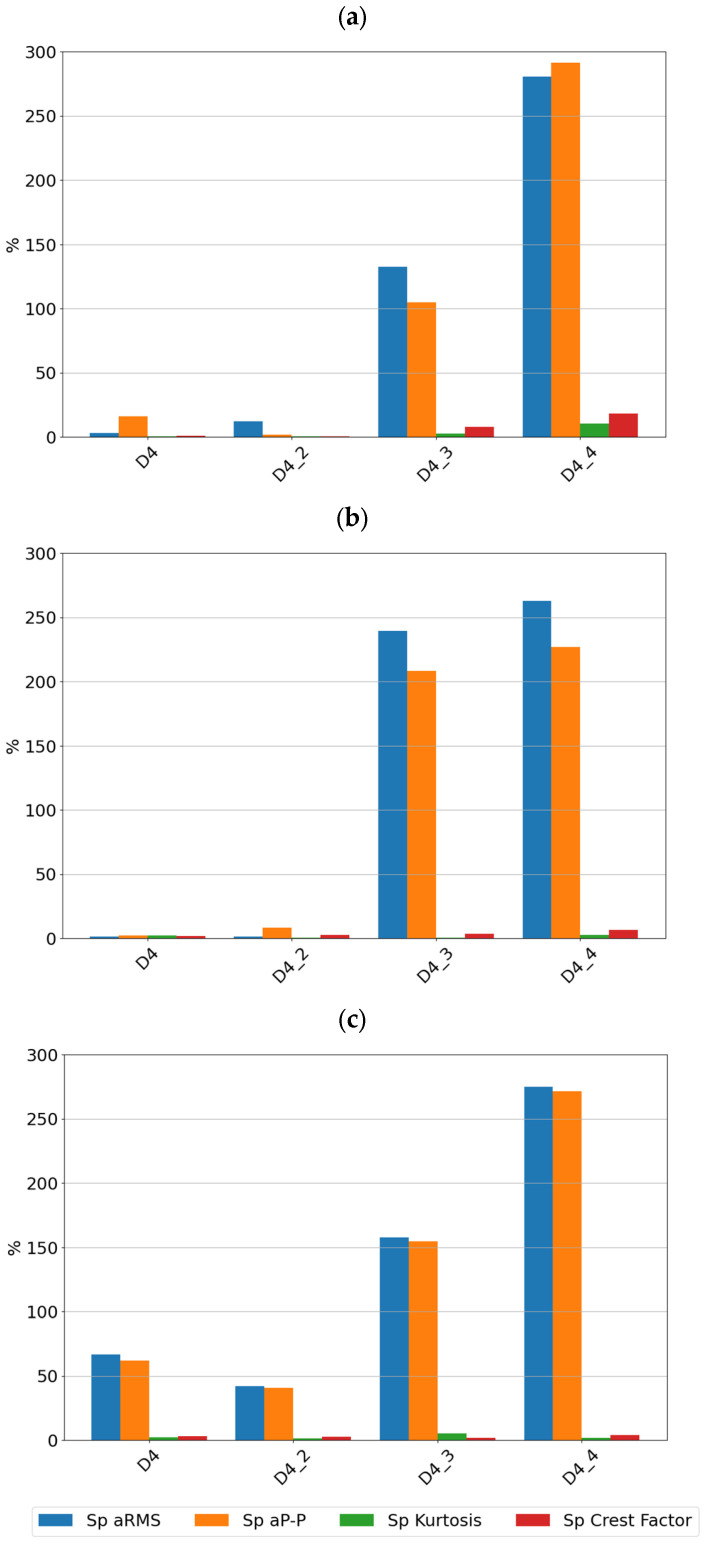
Sensitivity values for different stages of cage damage for sensor SE1 at speeds of (**a**) 600 rpm, (**b**) 1500 rpm, and (**c**) 3000 rpm.

**Figure 10:**
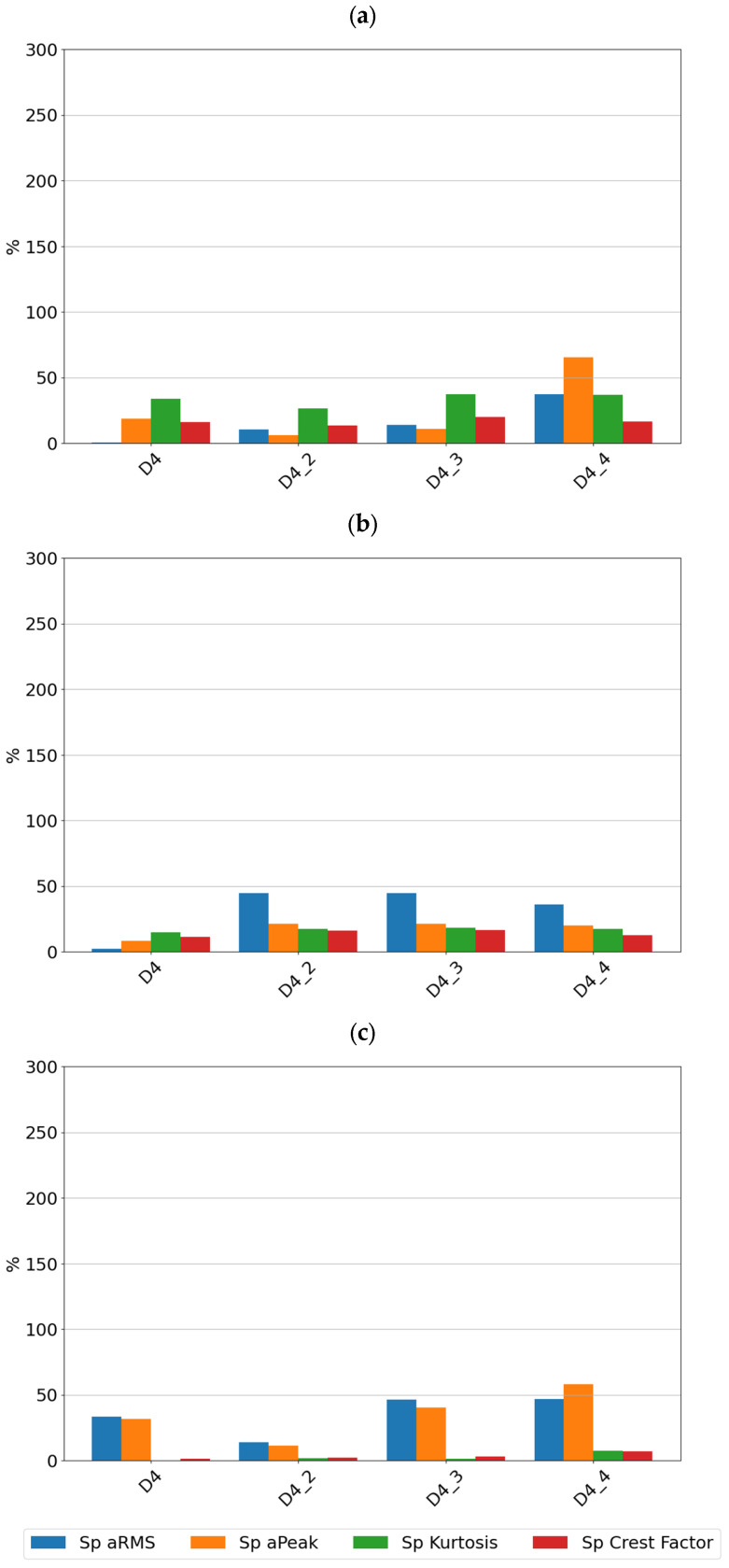
Sensitivity values for different stages of cage damage for sensor SE2 at speeds of (**a**) 600 rpm, (**b**) 1500 rpm, and (**c**) 3000 rpm.

**Figure 11:**
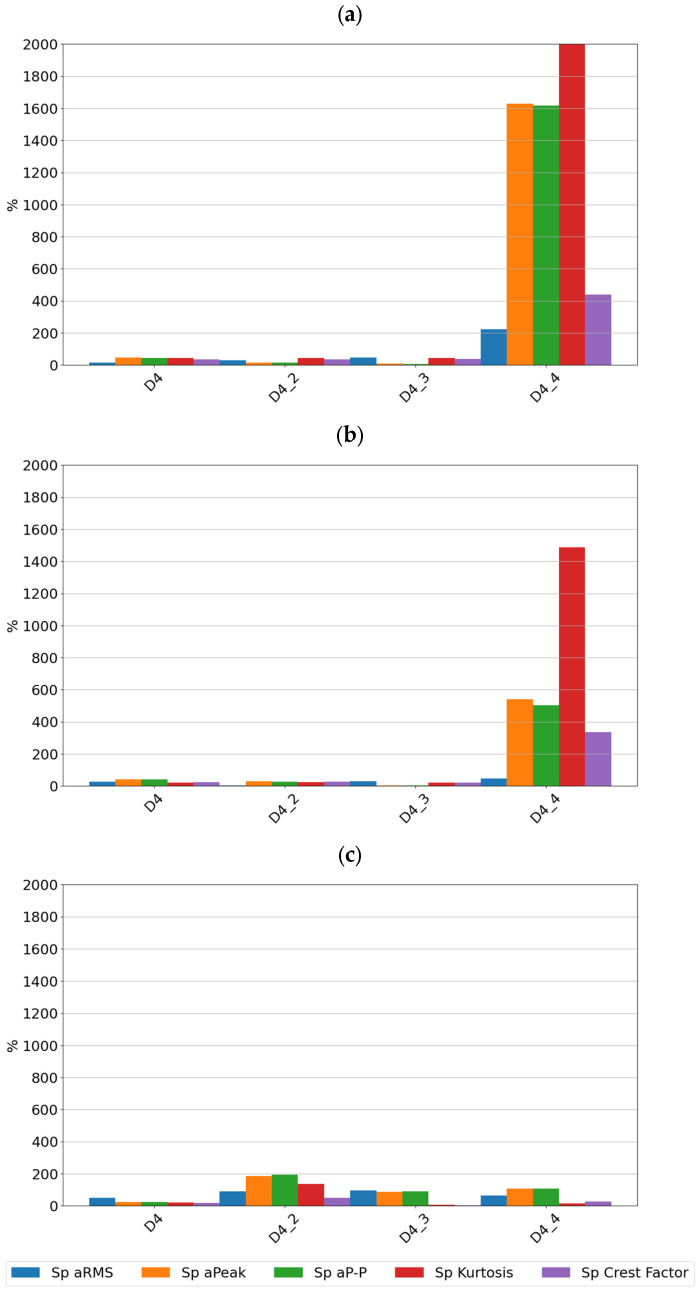
Sensitivity values for different stages of cage damage for sensor SEref at speeds of (**a**) 600 rpm, (**b**) 1500 rpm, and (**c**) 3000 rpm.

Figure 9, Figure 10 and Figure 11 show the sensitivity plots of the feature values for the different stages of cage damage considered.

It can be seen that for low-intensity cage damage (D4—single break; D4_2—two breaks), the sensitivity for both sensors does not exceed 100%. In contrast, high-intensity cage damage is best detected on the basis of peak and rms amplitudes determined over the full frequency range. The peak and peak-to-peak values of the accelerations are also a diagnostic parameter that characterizes this type of damage well.

From the sensitivity values point of view, it was observed that the sensitivity increases with increasing speed, which is the expected effect for bearings, but in the case of cage damage, no significant increase in sensitivity with speed was observed.

**Figure 12:**
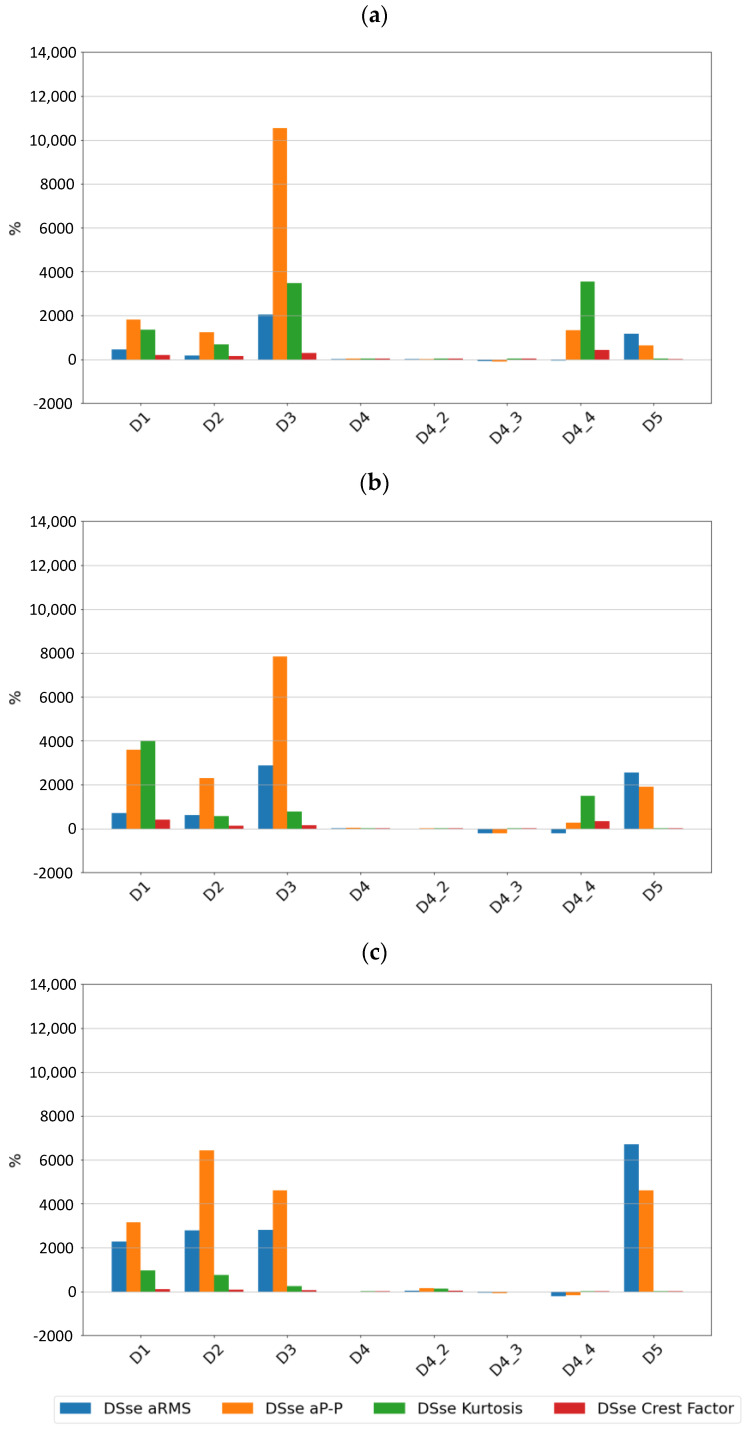
Differential sensitivity value for sensor SE1 at speeds of (**a**) 600 rpm, (**b**) 1500 rpm, and (**c**) 3000 rpm.

**Figure 13:**
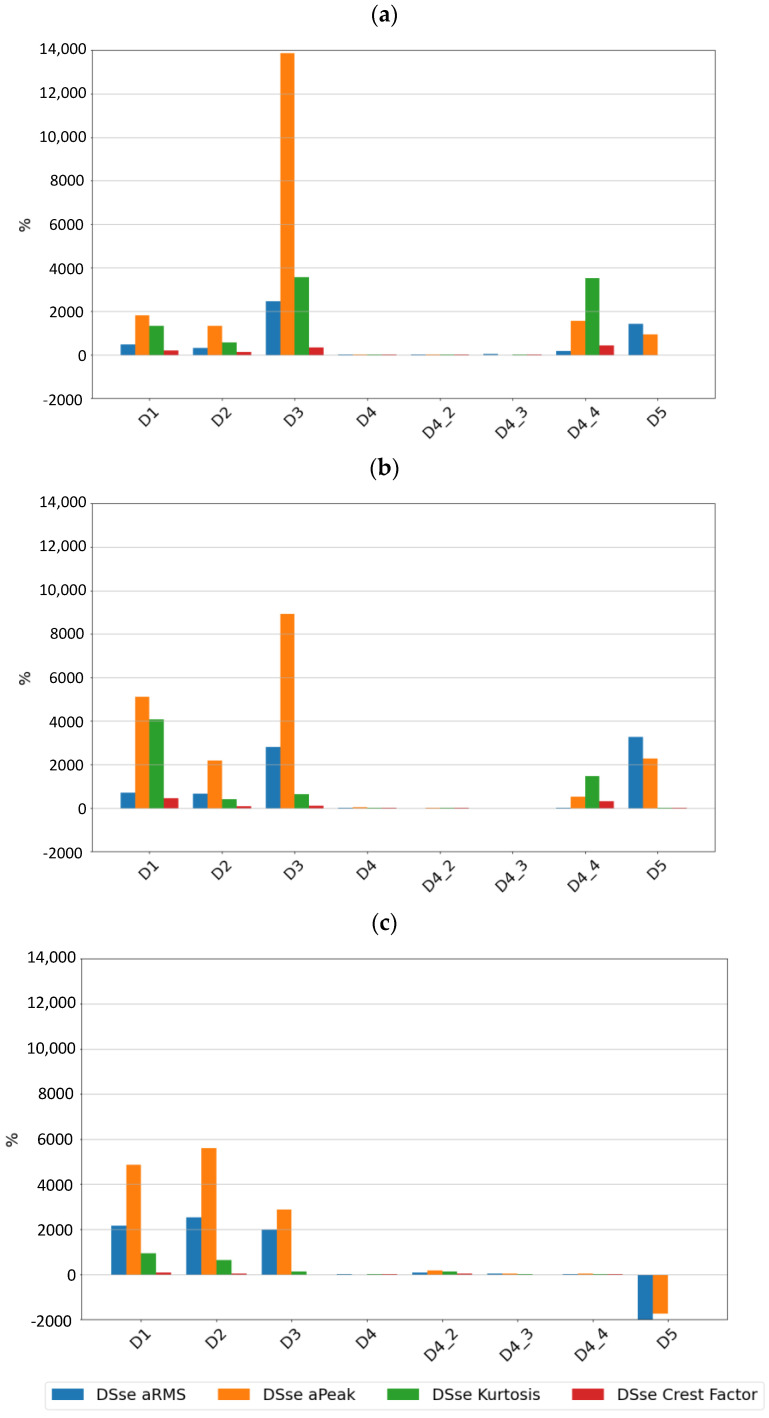
Differential sensitivity value for sensor SE2 at speeds of (**a**) 600 rpm, (**b**) 1500 rpm, and (**c**) 3000 rpm.

If one relates the values of the sensitivity of the features of the vibration signals of the tested sensors SE1 and SE2 to the features calculated for signals from the piezoelectric sensor by analyzing the values of the differential sensitivity SD presented in Figure 12 and Figure 13, it is easy to see that in most cases, the values are positive, and for typical bearing damage, the values are very high, which indicates that in the case of rolling element bearings, the determination of the signal features in a wide frequency range allows for the early detection of typical defects related to the material fatigue of the races and rolling elements. In the case of a lack of lubrication and cage damage, the results do not clearly indicate an advantage for the piezo sensor; in which case, for example, sensor SE2 had better sensitivity to a lack of lubrication and sensor SE1 greater sensitivity to intensive cage damage at 3000 rpm.

When comparing the differences in the sensitivity of the signal features between the piezoelectric sensor and the digital sensors, it was noted that for the piezoelectric sensor, kurtosis was a much more sensitive parameter. In the case of typical bearing failures, kurtosis did not show high sensitivity in the case of the SE1 and SE2 sensors, which may be due to the way in which it is estimated, which is not completely known as far as the SE1 and SE2 sensors are concerned.

## 4. Conclusions

On the basis of the research carried out, it can be concluded that commercial digital vibration sensors are sensitive to basic rolling bearing damage of medium and high intensity, which makes it possible to detect bearing faults and prevent unexpected machine failures, provided that the correct warning and alarm thresholds are set in condition monitoring systems and that maintenance services respond correctly to the emergence of alarm signals. The sensitivity of sensor bearing fault detection is dependent on the frequency band. The higher the sensor’s processing capabilities in the higher frequency range, the higher the sensitivity to even low-intensity damage increases, as can be seen from a comparison of the two sensors SE1 and SE2, in which the diagnostic parameters of the vibration signals were determined in the bands 2–3500 Hz and 1000–4000 Hz, respectively. This fact is also confirmed by comparing the sensitivity of the signal features of digital sensors with a piezoelectric sensor, whose upper frequency of the measurement range was 10,000 Hz.

It can also be argued that the high diagnostic sensitivity values for the piezoelectric sensor are due to the nature of the sensor’s operation; however, at this stage of the research, it is not possible to state unequivocally that the use of a piezoelectric transducer increases the diagnostic sensitivity to a decisive degree compared to MEMS capacitive accelerometers. This would require a comparison of accelerometers in similar processing bands, which will be the subject of the authors’ further research.

Research shows that diagnostic sensitivity depends on the frequency band as well as the type of damage. It can be assumed that these two factors determine the ability of sensors to detect various bearing defects. To investigate these relationships, it is necessary to conduct broader studies on a wider statistical sample and an expanded number of vibration sensors. This will be the subject of further research by the authors.

## Figures and Tables

**Table 1:** Classification of bearing condition according to the ranges of the parameter $K\left(t\right)$.

$K\left(t\right)$	Classification of Bearing Condition
>1	Condition improvement
1.0–0.5	Standard bearing condition
0.5–0.2	Deteriorating bearing condition
0.2–0.02	Advanced damage
<0.02	Failure

**Table 2:** Comparison of parameters of exemplary digital vibration sensors based on MEMS accelerometers.

	Balluff BCM0002	Banner QM30VT2	Sick MPB10
Number of axes	3	2	3
Measuring range	±16 g	N/A	±8 g
Measuring range vRMS	N/A	0–46 mm/s	0–100 mm/s (at 88 Hz)
Frequency range	2–2500 Hz	10–4000 Hz	0.78–3200 Hz
Accuracy	±10% (2–1800 Hz) ±3 dB (2–2500 Hz)	±10% (at 25 °C)	±6%
Interface	IO-Link 1.1	RS-485 (Modbus RTU)	IO-Link 1.1
Operating temperature	−25 to +70 °C	−40 to +105 °C	–40 to +80 °C

**Table 3:** Comparison of diagnostic parameters estimated on vibration signals by digital vibration sensors.

Balluff BCM0003	Banner QM30VT2		Sick MPB10
	10–1000 Hz	1000–4000 Hz	
RMS Peak to Peak Max Kurtosis Crest Factor Skewness	vRMS (mm/s) vPeak (mm/s) aRMS (G) vPeak Component Frequency (Hz) Simplified Order Spectrum	aRMS (G) aPeak (G) Kurtosis Crest Factor	aRMS vRMS Variance Skewness Peak to Peak Shape factor Crest factor Impulse factor FFT spectrum analysis

**Table 4:** Condition classification of investigated bearings.

Bearing ID	Condition Characterization
D1	Damaged outer race
D2	Damaged inner race
D3	Damaged rolling element
D4	Bearing cage damaged (1 crack)
D4_2	Bearing cage damaged (2 cracks)
D4_3	Bearing cage damaged (3 cracks)
D4_4	Bearing cage missing
D5	No bearing lubricant

**Table 5:** Comparison of CV values for the considered vibration signal features estimated for new bearings in perfect condition.

Speed	600			1500			3000		
Feature Name	SEref	SE1	SE2	SEref	SE1	SE2	SEref	SE1	SE2
aRMS	3.25	4.66	-	3.51	7.52	-	2.7	6.22	-
aRMS HF	3.18	-	8.05	3.43	-	7.61	2.82	-	9.18
aPeak HF	14.19	-	19.96	15.56	-	14.6	15.42	-	15.86
aPP	14.48	11.15	-	14.50	13.02	-	16.34	9.12	-
K	9.11	12.51	19.92	7.59	7.53	9.48	17.49	5.33	6.39
CF	12.83	10.85	15.16	13.74	9.17	12.32	14.07	8.48	10.88

## Data Availability

The data presented in this study are available on request from the corresponding author.
